# The role of erythrocytes and erythroid progenitor cells in tumors

**DOI:** 10.1515/biol-2022-0102

**Published:** 2022-12-15

**Authors:** Hao Zhang, Guang-zhi Wan, Yu-ying Wang, Wen Chen, Jing-Zhi Guan

**Affiliations:** Department of Oncology, The Fifth Medical Center, Chinese PLA (People’s Liberation Army) General Hospital, Beijing 100091, China; Department of Oncology, The Eighth Medical Center, Chinese PLA (People’s Liberation Army) General Hospital, Beijing 100071, China; Postgraduate Department of Hebei North University, Zhangjiakou 075000, China; Department of Oncology, First Medical Center, Chinese PLA (People’s Liberation Army) General Hospital, Beijing, China; Department of Pathology, The Eighth Medical Center, Chinese PLA (People’s Liberation Army) General Hospital, Beijing 100091, China

**Keywords:** tumor, erythroid progenitor cells, immunotherapy, anemia, EPCs

## Abstract

In the current research context of precision treatment of malignant tumors, the advantages of immunotherapy are unmatched by conventional antitumor therapy, which can prolong progression-free survival and overall survival. The search for new targets and novel combination therapies can improve the efficacy of immunotherapy and reduce adverse effects. Since current research targets for immunotherapy mainly focus on lymphocytes, little research has been done on erythrocytes. Nucleated erythroid precursor stem cells have been discovered to play an essential role in tumor progression. Researchers are exploring new targets and therapeutic approaches for immunotherapy from the perspective of erythroid progenitor cells (EPCs). Recent studies have shown that different subtypes of EPCs have specific surface markers and distinct biological roles in tumor immunity. CD45^+^ EPCs are potent myeloid-derived suppressor cell-like immunosuppressants that reduce the patient’s antitumor immune response. CD45^−^ EPCs promote tumor invasion and metastasis by secreting artemin. A specific type of EPC also promotes angiogenesis and provides radiation protection. Therefore, EPCs may be involved in tumor growth, infiltration, and metastasis. It may also be an important cause of anti-angiogenesis and immunotherapy resistance. This review summarizes recent research advances in erythropoiesis, EPC features, and their impacts and processes on tumors.

## 1 Introduction

The targeted tumor microenvironment (TME) has been recognized as a promising cancer surveillance and treatment approach in recent years [1,2]. The body’s internal environment influences the TME but is also adjusted and re-edited by the TME [3]. The TME includes tumor cells, peripheral immune cells, neovascularization, endothelial cells, fibroblasts, and extracellular matrix [4]. Erythroid progenitor cells (EPCs) in the TME can suppress T cells by producing reactive oxygen species (ROS), interleukin (IL)-10, and transforming growth factor β (TGF-β) in a paracrine and intercellular manner. Their ability to suppress T-cell immunosuppression is more robust than myeloid-derived suppressor cells (MDSCs) [5,6]. Erythrocytes are the most abundant blood cells in the peripheral blood, providing a stable supply of oxygen and an essential part of the immune system [7,8]. In addition, reduced erythrocyte volume can lead to anemia, which is common in patients with advanced malignancy. Numerous clinical studies have shown that anemia is an independent risk factor for poor prognosis in tumor patients. Therefore, erythroid cells and tumors are closely related. Since mature erythrocytes do not have a nucleus, they are almost unaffected by extracellular factors. It is difficult for medical interventions to rapidly and effectively influence erythrocytes. EPCs are the progenitor stem cells of the red lineage and play an essential biological role in tumor progression. Therefore, researchers have explored the relevance of EPCs to tumors, aiming to provide new targets for diagnosing and treating malignant tumors from the perspective of the erythroid lineage.

## 2 The role of erythroid cells in tumor immunity

As an essential component of the immune system [7,8], erythrocytes express various immune-related molecules and are involved in many immune responses and immune regulation. They also play an essential role in tumor immunity. Human erythroid complement receptor 1 (CR1) [9] on the surface of erythrocytes binds to C3b for immune adhesion clearance, prophagocytosis, and lymphocyte regulation. Erythrocytes also produce cytokines or specific signaling molecules to regulate the immune response. Erythroid cells form rose junctions with invasive pathogens (including tumor cells and bacteria) and facilitate their elimination as macrophages pass through the liver and spleen. Erythroid cells re-enter the bloodstream [10,11]. Thus, the immunity mediated by erythrocytes may prevent cancer cells from spreading through the bloodstream.

In addition, erythrocytes contain antioxidants that reduce the autotoxic effects of free radicals released by phagocytes during phagocytosis, and it also increases the phagocytic effect of phagocytes [12]. Erythrocytes express the Duffy chemokine antigen/receptor (DARC), which removes angiogenic chemokines from the prostate TME and thus regulates prostate tumor growth [13]. Consequently, erythrocytes can bind cytokines released by tumor cells [14]. Healthy human erythrocytes promote T-lymphocyte proliferation and protect lymphocytes from apoptosis [15]. CD58 on the erythrocyte membrane can interact with CD2 on the lymphocyte membrane to induce cytokine production by T lymphocytes and indirectly promote B lymphocyte proliferation and differentiation [16,17]. These results demonstrate the critical role of erythrocytes in regulating the immune system [14]. The cytoplasm of erythrocytes contains the Natural Killer Enhancing Factor (NKEF), which significantly enhances the killing effect of NK cells on K562 cells [18].

**Figure 1:**
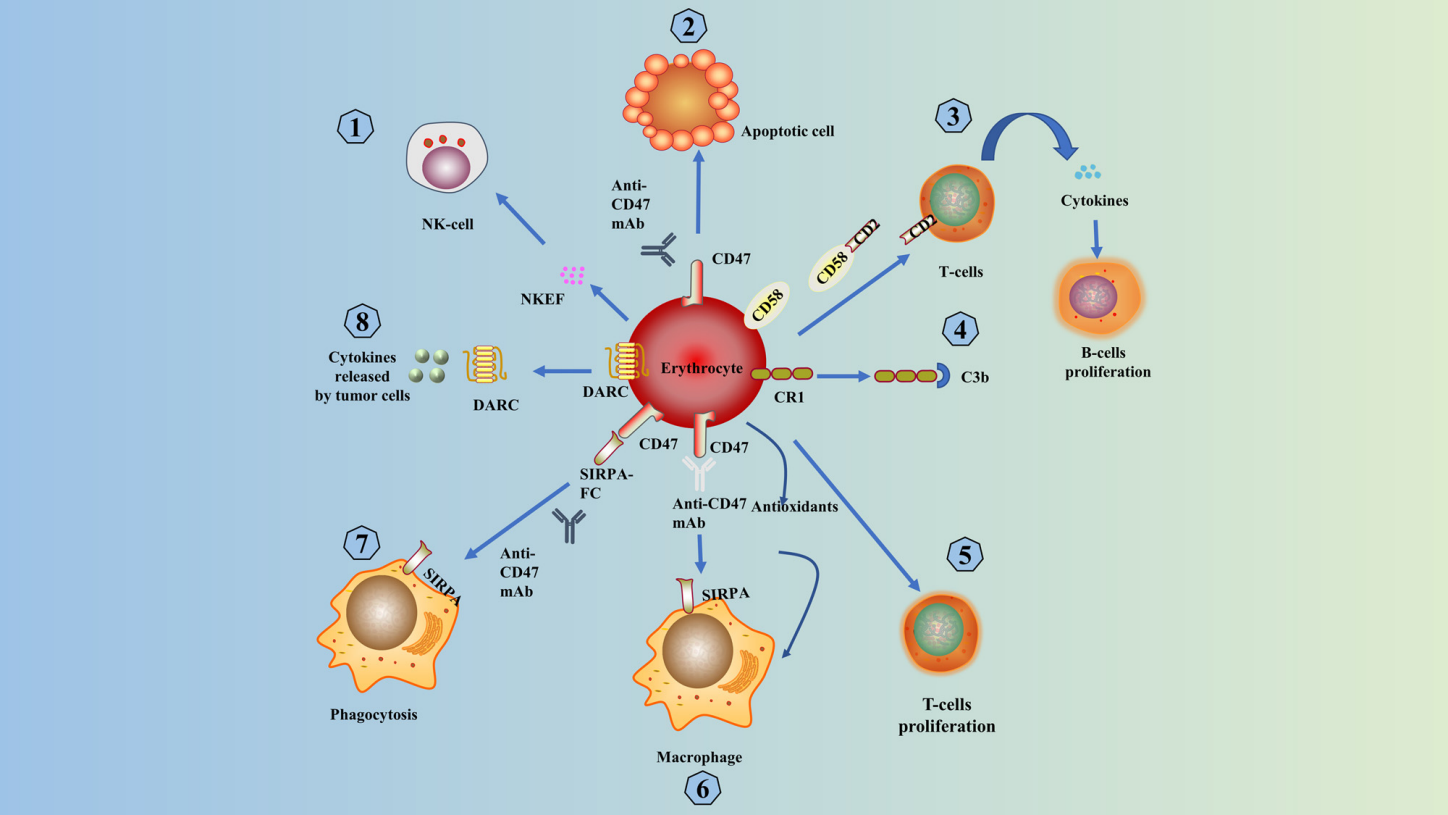
Primary mechanisms of erythrocyte-mediated immunity: (1) NKEF in erythrocyte cytoplasm enhances the killing ability of NK-cells; (2) erythrocytes with CD47 unbound anti-CD47 mAb show apoptosis; (3) CD58 on the erythrocyte membrane can interact with CD2 on the lymphocyte membrane, thus inducing cytokine production by T lymphocytes and promoting the proliferation and differentiation of B lymphocytes; (4) erythrocytes complete IAC through CR1 binding to C3b; (5) erythrocytes in healthy humans can promote T-lymphocyte proliferation; (6) antioxidants contained in erythrocytes increase phagocytosis; (7) signal regulatory protein α interacts with CD47 to also promote phagocytosis of apoptotic cells [23]; and (8) erythrocytes expressed DARC can bind and scavenge cytokines released by tumor cells.

Previous studies have found that cancer cells alter the cytokine profile and immune function of erythrocytes. Researchers exposed erythrocytes to non-small-cell lung cancer cell lines to change the erythrocytes and their cytokine profile. It was found that in the presence of these altered erythrocytes, T-cell proliferation was stimulated to a more significant extent and was no longer protected by stimulus drive. It is driven to release a variety of cancer-related cytokines [14]. The multiple immune mechanisms of erythrocytes protect them from suppressive or adverse host responses, and erythrocytes are suitable as carriers for drug delivery [19]. Several oncology drugs delivered via nano-erythrocytes have shown promising antitumor efficacy [20,21,22] (Figure 1).

## 3 Cancer-associated anemia

Anemia is one of the most common complications of cancer, with 30–90% of cancer patients suffering from anemia [24]. Among hematologic tumors, the rate of anemia is high, and clinical studies have shown that approximately 45% of lymphoma patients develop anemia [25]. There is a rich network of blood vessels in the gastrointestinal mucosa or lung tissue. Cancer cells can destroy these blood vessels early, leading to chronic blood loss. Therefore, lung cancer, gastric, and early-stage colon cancer patients may have combined anemia. The prognosis is often poor. Many long-term clinical studies have shown that patients with malignant tumors present with anemia [26]. Statistically, patients with anemia have shorter survival times and a 65% increased overall risk of death compared to cancer patients without anemia [27]. Their quality of life and prognosis is significantly worse [28]. For instance, Caro et al. have found anemia substantially decreases the overall survival of patients with nasopharyngeal carcinoma and increases the risk of tumor metastasis [27].

### 3.1 Mechanisms of anemia in cancer patients

There are two forms of iron deficiency in cancer patients: absolute iron deficiency (AID) and functional iron deficiency (FID). Among more than 40% of oncology patients, FID is present [29]. AID is characterized by depleted iron stores and inadequate iron supply, whereas FID has adequate iron stores but insufficient iron supply to support erythropoiesis and other iron-dependent metabolic pathways [30,31]. FID is a significant cause of anemia in chronic disease, also known as cancer anemia or cancer-related anemia [32,33]. The leading cause of FID in cancer is the release of pro-inflammatory cytokines associated with cancer, such as IL-6, IL-1, tumor necrosis factor α (TNF-α), and interferon-γ (IFN-γ). These cytokines increase the synthesis of hepcidin, which decreases the amount of iron released into the bloodstream [32]. Myelosuppression by radiation, chemotherapy, and erythropoietin (EPO) stimulant agents for anemia can cause FID [30,32]. A common complication in cancer patients, chronic kidney disease, can cause FID by decreasing erythropoiesis and increasing hepcidin levels [34,35].

Patients with long-term tumors are often associated with chronic systemic inflammation, and multiple cytokines can lead to stalled maturation of early EPCs [36]. Tumor cells produce pro-inflammatory cytokines (e.g., TNF-α, IL-1, IL-6) that cause anemia, reduce erythrocyte lifespan, and alter energy metabolism by altering iron homeostasis, inhibiting erythropoiesis and blocking EPO synthesis, and affecting EPO activity [37]. It also inhibits EPO production and decreases the responsiveness of bone marrow hematopoietic stem cells (HSCs) and EPCs to EPO, leading to impaired erythropoiesis [38]. Recent studies have shown that TNF-α can upregulate PU.1 and GATA2 in HSPCs to inhibit erythroid differentiation and lead to anemia [39]. IFN-γ induces differentiation arrest in EPCs and shortens the lifespan of erythrocytes, which causes anemia [40]. In addition to the above-mentioned pro-inflammatory cytokines, elevated plasma VEGF concentrations stimulated increased EPO secretion and elevated circulating reticulocyte indices, which promote early EPC expansion in the bone marrow and spleen [41].

### 3.2 Mechanisms of the pro-tumor effect of anemia

The specific mechanism of anemia’s pro-tumor effect is unknown, and it may be multifaceted. Hypoxia may be one of the essential mechanisms. First, hypoxia may alter the intra- and extracellular distribution of chemotherapeutic drugs, enhance the expression of various drug resistance genes, and reduce the sensitivity of tumor cells to radiotherapy; second, hypoxia may activate angiogenesis, which enhances the invasiveness and metastatic risk of tumors and increases tumor survival. Furthermore, hypoxia promotes the production of cytokines and chemokines, which recruit tumor-promoting immune cells and weaken the tumor immune response [42,43,44]. All of the mechanisms mentioned above affect the treatment of tumors. Some studies have also found that anemia can increase the production of various growth factors and matrix-degrading enzymes and stimulate angiogenesis. The new blood vessels can provide nutrients and oxygen for tumor growth, the main pathway for tumor invasion and metastasis. Anemia can also impair immune function, weakening the anti-tumor immune response and significantly reducing the patient’s ability to defend against pathogenic infections. Studies have shown that the percentage of EPCs in peripheral blood is significantly increased in malignant tumor patients with anemia. EPCs in peripheral blood may promote tumor progression and metastasis by reducing immunity and releasing specific cytokines. We, therefore, consider that promoting the differentiation of EPCs, treating anemia, and reducing ineffective erythropoiesis may be a therapeutic strategy used to restore anti-tumor immunity and lessen the pro-tumor effects of EPCs.

## 4 The role of EPCs in tumors

The first report on tumors and EPCs was published in the 1980s by Geissler et al. They found that the percentage of EPCs in peripheral blood was significantly increased in patients with acute and chronic lymphocytic leukemia [45]. EPCs in peripheral blood increased dramatically in patients with combined anemia and negatively correlated with hemoglobin [46]. This may be due to the direct inhibition of erythroid differentiation by TME or some cytokines from tumor cells [46], as well as the significant decrease in EPO concentration in tumor patients, which blocks the differentiation pathway from EPCs to erythrocytes [46], increasing EPCs in peripheral blood and a decrease in mature erythroid cells. Hence, tumor patients present with anemia at the same time.

### 4.1 Surface markers of EPCs

Although EPCs were defined as early as 1970, few studies have detailed their cellular and molecular characteristics. According to the available studies, EPCs can express some surface markers, including IL-3R, FLT3, MPL, CD36, CD41, CD71, CD105, and CD235a [6,46,47,48,49,50]. After EPCs mature, CD71 [51] and CD45 [52] also disappear. Cells with CD71^+^CD235a^+^ and CD71^+^TER119^+^ have been defined by researchers as EPCs in human and mouse peripheral blood and spleen [46,47].

### 4.2 The role of CD45^+^ EPCs in tumors

In recent years, the role of EPCs in tumorigenesis and progression has attracted the attention of researchers. Research has reported an immunosuppressive cell of red lineage origin, CD45^+^ EPCs (CD45^+^CD71^+^CD235a^+^), similar to MDSCs [53], regulatory T cells (Tregs) [54], and tumor-associated macrophages [55]. CD45^+^ EPCs were significantly increased in patients with advanced tumors combined with anemia in peripheral blood. CD45^+^ EPCs produce arginase-2 [56], IL-10, and ROS [5], which inhibit T-cell proliferation and production of IFN-γ [57]. CD45^+^ EPCs also function as immunosuppressors in a direct cell-to-cell contact and paracrine manner. This has caused a decrease in antiviral, antibacterial, and antitumor immune responses in tumor patients and has contributed to tumor progression. Subsequently, researchers systematically analyzed the composition of immune cells in the spleen of tumor-bearing mice. They found that, in addition to elevated MDSCs and Tregs, EPCs were abundantly accumulated in the spleen. In mice bearing a tumor and aged 21–28 days, EPCs accounted for as much as 50% of the total spleen cell composition [46]. Moreover, EPCs expanded in the spleen, peripheral blood, and liver of tumor-bearing mice and cancer patients [5]. CD45^+^ EPCs can infiltrate murine and human tumors, and their abundance in TME is much higher than that of MDSCs or Tregs [5,6,46], and CD45^+^ EPCs also inhibit T cells more than MDSCs [5].

Previous studies have suggested that the spleen is the organ of origin of CD45^+^CD71^+^CD235^+^ EPCs. Hepatocellular carcinoma (HCC) could be a circulating origin or produced directly in HCC tissues [5]. The higher abundance of CD45^+^CD71^+^EPCs in HCC tissues, the more important their immunosuppressive capacity is, and they are closely associated with multiple prognostic factors. Therefore, immunofluorescence screening of CD45^+^CD71^+^ EPCs in tumor tissues may be a new clinical method to predict tumor recurrence after radical surgery for HCC [5].

The rapid proliferation of tumor cells leads to local hypoxia of the microenvironment. Under hypoxic conditions, CD45⁺ EPCs can regulate lipid metabolism and enhance energy metabolism in lymphoma cells via the AMPK–ACC–CPT1A pathway, which further promotes cell proliferation and inhibits apoptosis in lymphoma cells. Animal experiments have also shown that transplantation of CD45⁺ EPCs significantly increased resistance to antiangiogenic drugs, and ROS played a vital role in these processes. Therefore, CD45^+^ EPCs in lymphoma may enhance resistance to antiangiogenic drugs in tumors through ROS [58].

### 4.3 The role of CD45^−^ EPCs in tumors

An EPC (also called Ter cells) with a phenotype of CD45^−^Ter119^+^CD71^+^ is enriched in the enlarged spleen of the cancerous host; CD45^−^ EPCs are more mature EPCs than CD45⁺ EPCs. Since CD45^−^ EPCs have no direct inhibitory effect on CD4^+^CD8^+^ T cells [5], they do not promote tumor progression by inhibiting antitumor immunity. It promotes tumor invasion and metastasis through the secretion of artemin into the blood [59,60,61] as artemin promotes the proliferation of surviving cancer cells and increases tumor cells’ invasiveness [59,62,63,64].

Recent studies have shown that increased serum artemin concentration and receptor expression are associated with poor prognosis in cancer patients. They have demonstrated that CD45^−^ EPCs can promote tumor progression and metastasis through artemin [62]. Patients with pancreatic ductal adenocarcinoma have higher cell counts of CD45^−^ EPCs in the spleen than patients with non-cancerous pancreatic tumors or benign pancreatic masses. Splenic CD45^−^ EPCs activate the GFRα3-ERK signaling pathway by expressing artemin to promote PDAC cell proliferation and invasion. High splenic CD45^−^ EPCs cell counts often indicate poor prognosis and are associated with tumor size and lymph node metastasis [62].

**Figure 2:**
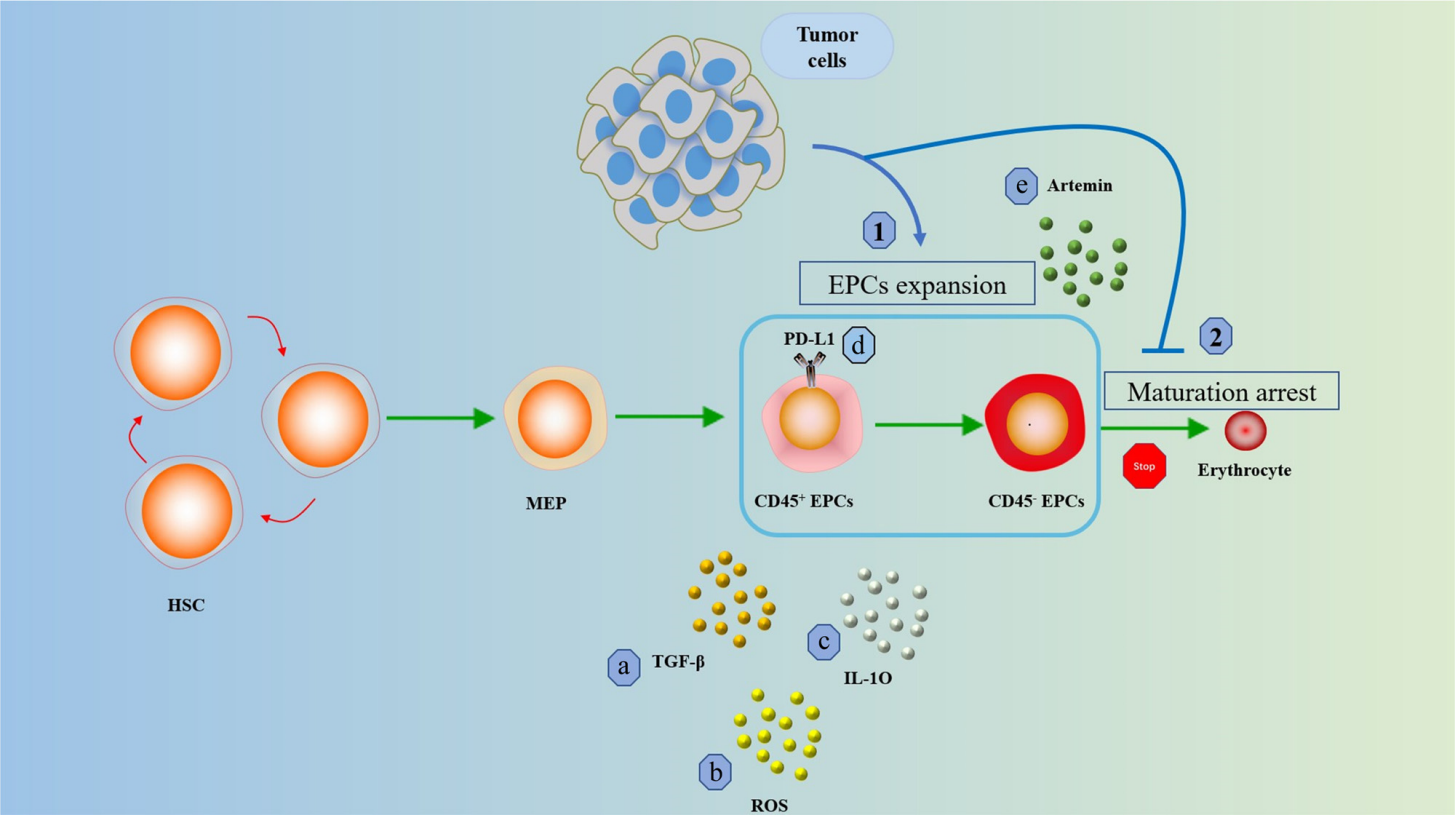
The role of EPCs in cancer. (1) Tumors promote the expansion of EPCs and (2) tumors inhibit the maturation and differentiation of EPCs. Early CD45^+^ EPCs use (a) TGF-β, (b) ROS, (c) IL-10 and (d) PD-L1 regulates immune responses. More mature CD45^−^ EPCs regulate cancer progression through (e) secretion of Artemin.

**Figure 3:**
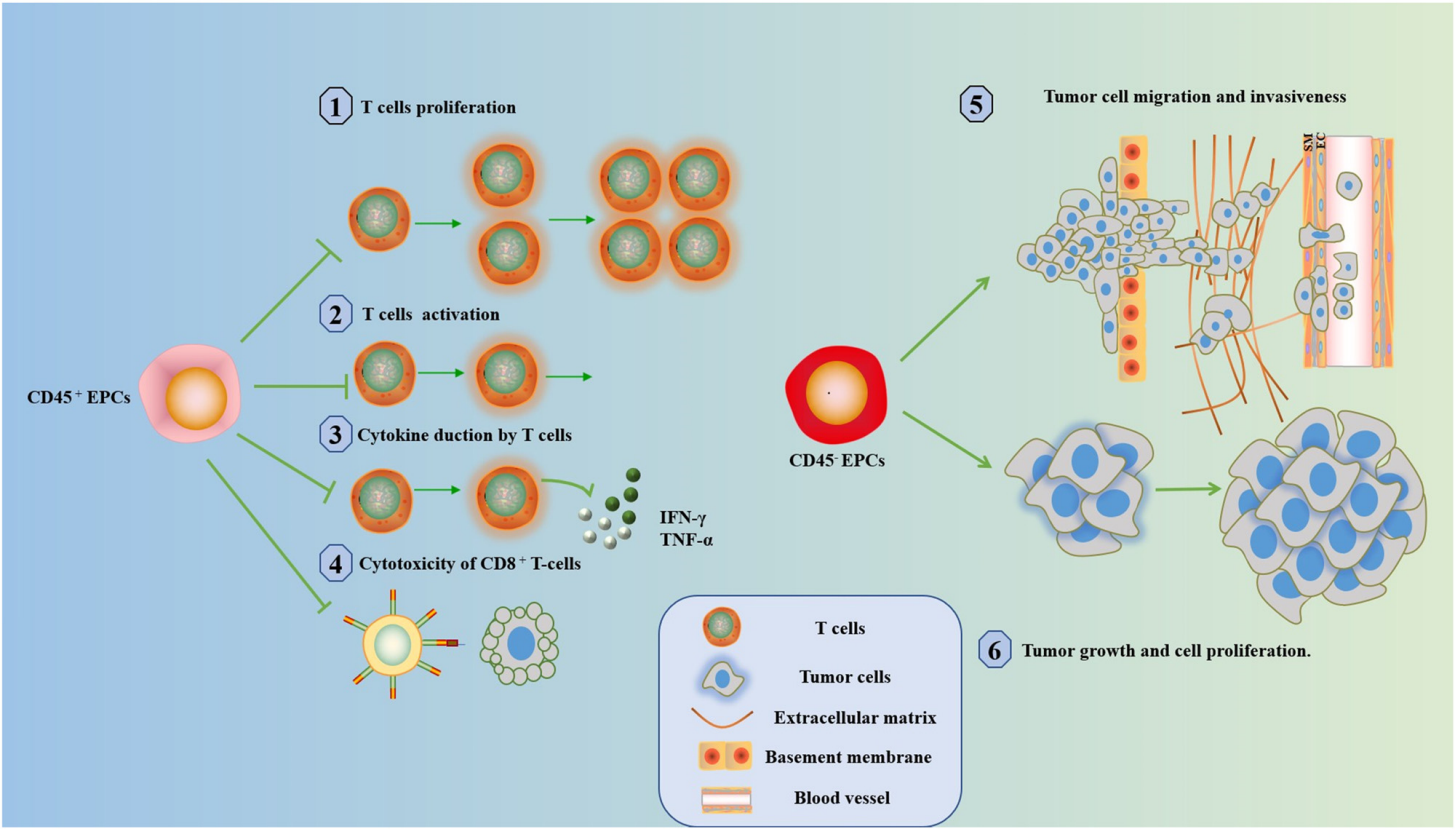
The role of EPCs n cancer. CD45^+^ EPCs inhibit (1) T-cell proliferation, (2) T-cell activation, (3) IFN-γ and TNF-α production, and (4) CD8^+^ T-cell cytotoxicity. CD45^−^ EPCs promote (5) tumor cell metastasis and invasion and (6) tumor growth and cell proliferation.

Until now, the clinical applications targeting Artemin and its signaling pathway are still relatively few. The specific mechanism by which artemin promotes tumor growth and distant metastasis also remains undetermined [59]. Han et al. found that inhibition of artemin secretion significantly hindered liver cancer growth and reduced the cancer-promoting ability of CD45^−^ EPCs. In addition, the study found that deletion of GFR-α3 and RET resulted in deficient artemin signaling, including reduced phosphorylation of AKT and ERK. This provides us with a new perspective on treatment and its signaling pathway to inhibit the tumor-promoting effect of CD45^−^ EPCs [65] (Figures 2 and 3).

### 4.4 The immunomodulatory properties of CD71^+^ EPCs disappear during their maturation

Since CD71^+^ EPCs inhibit T-cell proliferation and IFN-γ production via ARG and ROS, ROS, ARG1, and ARG2 were highest in the early stage of CD71^+^ EPCs, so the immunosuppressive ability of CD71^high^ CD235a^mid^ EPCs was strongest in the early stage. The immunomodulatory properties of CD71 EPCs were most effective in the earliest stage of their differentiation. With the differentiation and maturation of EPCs, CD71 on the surface of EPCs begins to decrease when it reaches Ortho-E and disappears after erythrocyte maturation. And the inhibitory effect of CD71^+^ EPCs on T cells is also completely lost, so the immunomodulatory properties of CD71^+^ EPCs are robust but transient, disappearing as they mature [56].

### 4.5 The radioprotective effect of CD31^+^ short-term EPCs

Platelet endothelial cell adhesion molecule-1 (PECAM-1/CD31) is a member of the immunoglobulin immunoreceptor tyrosine inhibitory motif superfamily [66]. HSC expresses CD31 from early embryonic stages to late adulthood. CD31^+^ Lin-c-kit^+^ Sca-1^−^ cells (CD31^+^ Sca-1^−^) can provide radioprotection, so CD31^+^ short-term EPCs may reduce the killing of tumor cells by radiotherapy [67].

## 5 Advances in the treatment of EPCs in tumors

With the in-depth research progress of EPCs, researchers began to explore the therapeutic value of EPCs. As early as 2007, Sasaki et al. exploited the property that EPCs can secrete angiogenic factors to promote angiogenesis in ischemic limbs [68]. Research by Mirmiran et al. using bifunctional transferrin (Tf) receptor one ligand-peptide, which targets the delivery of the characteristic gene into EPCs, was effective in treating the treatment of erythropoietic protoporphyria [69]. Wong et al. transformed CD36^+^ EPCs into a continuous cell line, CD36E. CD36E cells have EPC characteristics, of which approximately 27% of the cell population produces hemoglobin, which has potential application in tumors combined with anemia [70]. However, there are few experimental animal reports on tumor-targeted therapy for EPCs, and relevant clinical studies are still scarce.

In addition, Exosomes have been a hot research topic in recent years, and many exosome-based diagnostic and therapeutic approaches will soon enter clinical applications. Macrì et al. reported an EPC-derived vesicle (CD34^+^CD71^low^), which differed significantly between Diamond-Blackfan anemia (DBA) patients and the healthy population, may help to improve the diagnosis of DBA. Therefore, immunophenotypic analysis of extracellular vesicles derived from EPCs can be used to study the specific phenotype of erythroid differentiation [71].

Subsequently, Li et al. investigated the role of Danggui Buxue Tang in tumor therapy. They found that although Danggui Buxue Tang had no significant direct effect on tumor cell proliferation and apoptosis, it was able to promote the differentiation of EPCs and significantly attenuated the accumulation of EPCs, which enhanced the anti-tumor immune response and thus inhibited the progression of B16 melanoma [72]. This research provides a new strategy to inhibit malignant tumor progression and alleviate tumor-associated anemia treatment.

Hou et al. reduced tumor-induced CD45^−^ EPCs in the spleen of tumor-bearing mice by localized ionizing radiation (IR) tumors and thus decreased artemin secretion. However, subsequent experiments revealed that IR alone was unable to reduce the tumor-induced accumulation of CD45^−^ EPCs in the spleen of mice and required the mediation of type I/II IFN and CD8^+^ T cells. Intraperitoneally administered PD-L1 blocking antibodies also require the mediation of CD8^+^ T cells and IFN-γ to reduce the tumor-induced accumulation of CD45^−^ EPCs. Moreover, the therapeutic effect of tumor patients was associated with a decrease in CD45^−^ EPCs and artemin concentration. This research provides a new strategy for eliminating cancer-promoting EPCs [65].

Recently, Tan et al. reduced the frequency of EPCs in the spleen of an animal model of Lewis lung cancer by ultrasound-targeted microbubble and increased the frequency of T cells, especially CD8^+^ T cells. There was no pathological damage to the spleen, but this was insufficient to inhibit tumor progression. The researchers then combined UTMD with PDL-1 blockade treatment and found that the combination therapy inhibited tumor growth. Therefore, single immunotherapy and chemoradiotherapy may not be the best strategy for treating tumors. It is necessary to connect the value of targeted EPCs in enhancing immune response, improving the effectiveness of immunotherapy, and inhibiting tumor progression by finding appropriate treatment options [73].

## 6 Regulates differentiation and maturation of EPCs

With the established pro-tumor effects of EPCs, how to treat anemia and improve the prognosis of tumor patients will be a future research direction. Therefore, we believe that the pro-tumor effects of EPCs can be reduced by regulating erythropoiesis.

### 6.1 Erythropoiesis and its major regulatory pathways

Erythropoiesis is a complex process regulated by the interaction of multiple transcription factors and cellular molecules [74]. During this process, HSCs proliferate and differentiate into mature erythrocytes. HSC exists in a unique hematopoietic ecological niche [75]. In the early stages of erythropoiesis, HSCs differentiate into multipotent megakaryocyte-erythroid progenitor cells, followed by burst-forming unit erythrocytes (BFU-E) and colony-forming unit erythrocytes (CFU-E) [76]. During terminal erythropoiesis [77,78], CFU-E further differentiates into proto-erythrocytes (Pro-E). Pro-E decreases in volume, nuclei become concentrated, and they begin to produce specific proteins, such as hemoglobin, in large quantities. Pro-E then continues to differentiate according to the order of basophilic juvenile erythrocytes (Baso-E), poly-stained juvenile erythrocytes (Poly-E), and orthostained juvenile erythrocytes (Ortho-E). Finally, Ortho-E exits the nucleus and releases reticulocytes into the circulatory system [79]. It takes about 1 week for reticulocytes to mature into erythrocytes in healthy individuals [77]. As reticulocyte maturation proceeds, RNA, mitochondria, and ribosomes are degraded, and protein synthesis ceases [80]. Mature erythrocytes do not have a nucleus and have a biconcave disk shape. They have high concentrations of hemoglobin, which bind and transport O^2^.

The early stages of erythropoiesis are regulated by stem cell factor (SCF)/stem cell growth factor receptor (c-Kit), IL-3/IL-3 receptor (IL-3R) [81], and granulocyte-macrophage colony-stimulating factor (GM-CSF [81]) [77,82]. IL-3 regulates EPC self-renewal [83,84] and, together with activin A, induces mitosis and increases colony number [85]. Zinc finger transcription factor-2 (GATA-2), which is highly expressed in the early stages [86], promotes erythroid proliferation and inhibits erythroid differentiation [87,88]. Due to the switching mechanism of GATA, GATA-2 and GATA-1 are expressed at the early and late stages of erythropoiesis, respectively [86,89]. Zinc-finger transcription factor-1 (GATA-1) is erythropoiesis’s main transcriptional regulatory factor [90,91,92]. GATA-1 promotes erythroid differentiation in late erythropoiesis by positively regulating specific red lineage genes. GATA-1 also induces the expression of the anti-apoptotic protein Bcl-xL and erythropoietin receptor (EPO-R) together with transcription factor signaling and activator of transcription 5 (STAT5) [87,93]. During erythropoiesis, caspases cleave GATA-1 upon activation, blocking erythroid differentiation, and inducing cell death [94]. Chaperone heat shock protein 70 enters the nucleus after cystathionine activation and protects GATA-1 from caspase-3 cleavage, protecting early EPCs [94,95].

This phase of terminal erythropoiesis is dependent on iron metabolism and is regulated primarily by EPO [87,96] and SCF [81,97]. EPO binds to EPO-R and activates Janus kinase 2 (JAK2), and activation of JAK2 induces various signaling pathways, such as protein kinase B (AKT) and STAT5. This led to the activation of anti-apoptotic genes in the red lineage and enhanced the survival and proliferation of EPCs [98,99,100]. The binding between FAS and the FAS ligand (FASL) induces apoptosis in immature erythroid cells [87,101]. EPO interacts with membrane protein of death receptor family (FAS) and its ligand (FASL) to counteract the negative signal and prevent apoptosis of immature cells [102].

**Figure 4:**
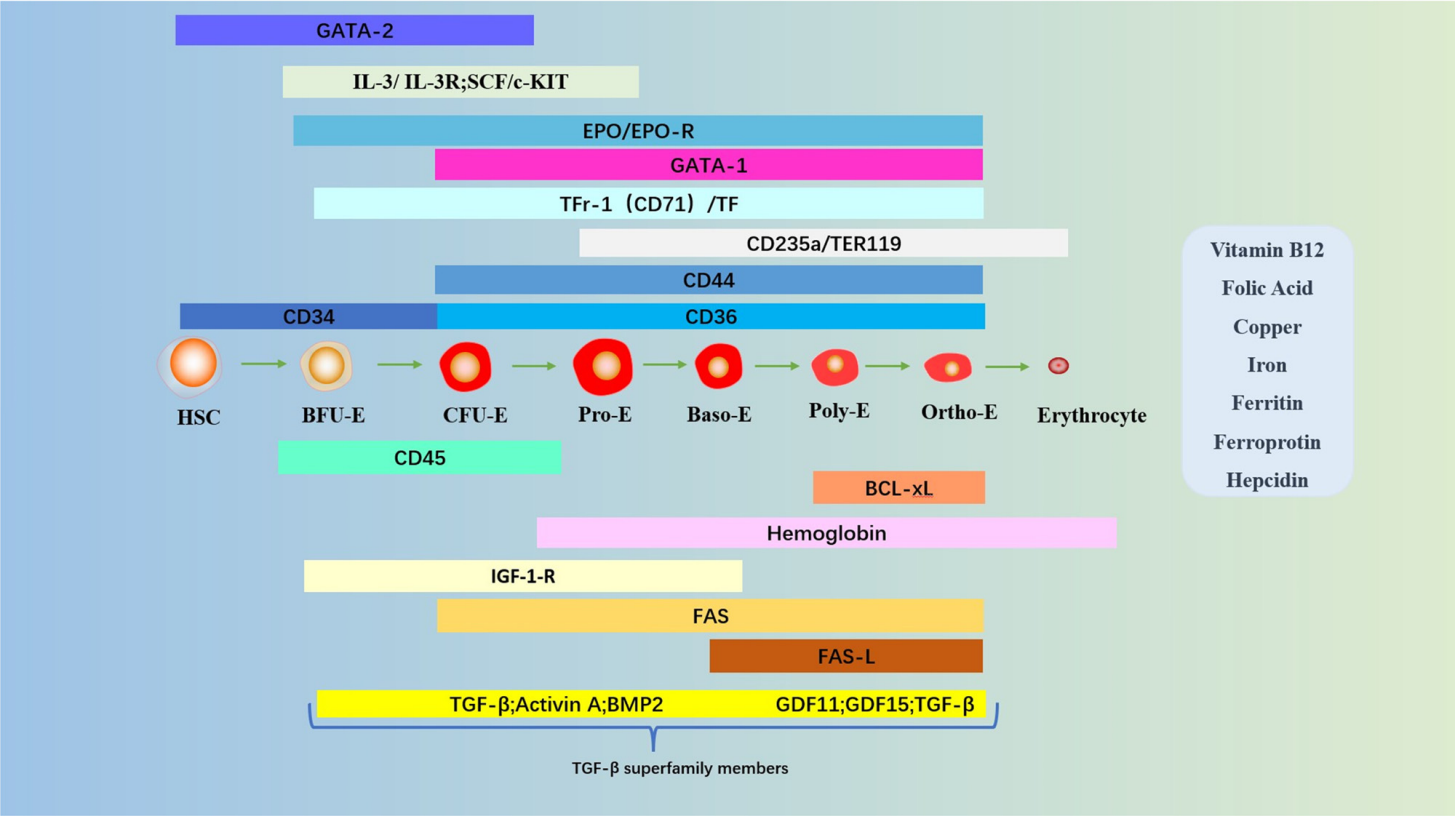
The main pathways and molecules involved in regulating erythropoiesis. The different stages are shown: HSC, BFU-E, CFU-E, Pro-E, Baso-E, Poly-E, Ortho-E, and erythrocytes. Molecules involved: zinc finger factors that bind GATA sequences (GATA-1, GATA-2); IL-3; IL-3-R; SCF; c-Kit; EPO; EPO-R; Ter-119, glycophorin A-associated protein; CD235a, glycophorin A; CD44, cell surface adhesion molecule; CD34, transmembrane phosphoglycoprotein; CD36, platelet glycoprotein protein 4; CD45, common marker of leukocytes; BCL-xL, anti-apoptotic protein; hemoglobin; FAS; FAS-L; Tf; TfR-1 (or CD71), transferrin receptor 1; TGF-β; activin A; BMP-2, bone morphogenetic protein 2; GDF, growth differentiation factor. Vitamins, trace elements, and iron metabolism proteins necessary for erythropoiesis: vitamin B12, folic acid, copper, iron, ferritin, ferroprotin, hepcidin).

In addition, vitamin B12 and folic acid, trace elements (copper and iron), and hepcidin are essential for erythroid maturation. Peroxisome proliferator-activated receptor alpha and glucocorticoid receptor synergistically promote self-renewal of EPCs [103]. Regulators of human erythroid cell expansion (RHEX) can promote EPC expansion and erythroid differentiation [104]. Erythroid differentiation and maturation are also negatively regulated by members of the Tf and its cellular receptor (Tfr) [105,106,107] and TGF-β superfamily [108]. Long-chain non-coding RNA regulates erythroid differentiation by coordinating with chromatin accessibility [109]. A complex network of transcription factors and epigenetic regulators regulates erythropoiesis [90,110]. Positive and negative regulation of erythropoiesis is essential for maintaining erythroid homeostasis [87] (Figure 4).

### 6.2 Promoting differentiation of EPCs into erythroid cells

As a result of dysregulated erythropoiesis in patients with advanced cancer, the expansion of EPCs effectively suppresses the antitumor immune response. Promote the differentiation of EPCs, reduce the production and accumulation of ineffective EPCs, and fundamentally reduce the inhibitory effect of EPCs on the immune response.

#### 6.2.1 The role of TGF-β in tumors

The TGF-β superfamily regulates late erythrocyte maturation through the SMAD signaling pathway. Dysregulation of this signaling pathway may impair erythroid maturation and ineffective erythropoiesis [111–113]. V-domain immunoglobulins that inhibit T-cell activation enable the cells to produce TGF-β repeatedly [113]. TGF-β signaling has a myelosuppressive effect, blocking EPCs’ proliferation and accelerating their differentiation [114]. The activation of TGF-β and Smad3 is essential for the tumor-induced formation of CD45^−^ EPCs in the spleen (also known as Ter cells) [62]. In TGF-β knockout tumor mice and Smad3-deficient mice, the expansion of CD45^−^ EPCs was significantly reduced [59]. Therefore, activation of TGF-β and Smad3 contributed to the formation of Ter cells, resulting in more ineffective erythropoiesis.

The TGF-β superfamily includes TGF-β, activin, BMP, and GDF-11, and these cytokines play an essential role in erythropoiesis [115]. In particular, activin and GDF-11 are inhibitory on advanced erythropoiesis [116]. TGF-β not only plays a pro-differentiation function in erythropoiesis but is also a master regulator of numerous cellular functions, including cellular immunity [117–119]. Activin-A and BMP immune responses through effects on resistant and non-immune cell populations [120].

In cancer, TGF-β can exert pro-tumorigenic effects through various mechanisms, including immunosuppression [121,122]. All advanced tumors overproduce TGF-β. TGF-β promotes tumor growth, invasion, and metastasis in an autocrine and paracrine manner and inhibits anti-cancer drug sensitivity [123,124]. Excess TGF-β also leads to the proliferation of EPCs. Inhibiting the SMAD signaling pathway could rescue the proliferation of T cells and IFN-γ production suppressed by EPCs [5]. Studies have shown that ligand capture fusion protein (ACE-536) binding to GDF11 effectively inhibited Smad2/3 signaling, reducing anemia and ineffective erythropoiesis in mice with myelodysplastic syndrome [111]. Another study found that RAP-011, similar to ACE-536, improved inadequate erythrocyte production and corrected anemia in mice [112]. Preclinical studies have shown that some anti-TGF-β immunotherapies are effective for oncology treatment, specifically when combined with immune checkpoint inhibitors [125,126]. Therefore, immunotherapies targeting TGF-β activation or signaling may inhibit the tumor-promoting effects of EPCs and the tumor-promoting effects of TGF-β, thereby improving the efficacy of immunotherapies for various cancers.

#### 6.2.2 The role of EPO in tumors

EPO maintains erythrocyte homeostasis by precisely regulating the number of erythrocytes through the degree of tissue oxygenation. Different levels of EPO also have various effects on erythropoiesis. When caspases are activated, cells with low sensitivity undergo apoptosis at low EPO levels. At mild EPO levels, cells are blocked or apoptotic during maturation, and at higher EPO levels, most cells survive and differentiate [127].

It was found that EPO-R-deficient mice develop severe anemia and impaired EPC function [128], making the role of EPO in erythropoiesis crucial. Malignant cells and stromal cells in the TME produce vascular endothelial growth factor, which stimulates EPO secretion by stromal cells in the spleen expressing platelet-derived growth factor-beta receptor [129], thereby increasing EPO production.

However, the mechanism by which EPO acts in cancer is controversial. On the one hand, studies in cell culture and animal models have shown that the EPO pathway promotes tumor cell activity, proliferation, metastatic potential, treatment tolerance, and intervention [130]. On the other hand, there are also studies showing that EPO has no direct stimulatory effect on tumor cell growth. EPO has no immediate stimulatory effect on tumor cell growth [131,132]. A recent study found that the application of anti-EPO antibodies to B16 tumor-bearing mice prevented the expansion of the red lineage. Treatment with this antibody also had some antitumor effect, slowing down the subcutaneous growth of B16 tumors. Thus, EPO and red lineage cells are new players in tumor–host interactions [6].

In addition, clinical trials have reported that recombinant human EPO to treat anemia in cancer patients during chemotherapy or radiotherapy increases cancer patient mortality. However, the mechanism was not precise [133,134]. A recent study reported that EPO enhanced the immunosuppressive effect of CD45^−^ EPCs, which is detrimental to the antitumor development induced by local IR and blockade of programmed death-ligand 1 (PD-L1) [65], so EPO might indirectly affect T-cell function via Ter cells and their artemin production. Therefore, immunotherapies targeting EPO and its downstream signaling pathways could be strategies to reduce antitumor immunity.

#### 6.2.3 Other regulations that promote EPC differentiation

Notch signaling pathways play critical roles in the proliferation, development, maintenance, and differentiation of stem cells and multicellular organisms [135] and link between angiogenesis and self-renewal of CSCs [136]. Previous studies have reported that γ-secretase inhibitors (GSI), which inhibit Notch signaling, induce erythroid differentiation, and promote hemoglobin production in erythroid leukemia cell lines [137]. Therefore, the combination of GSI with anti-cancer drugs may be a promising strategy for cancer treatment [136,138].

Oxygen plays a critical role in erythropoiesis, and hypoxia promotes the loss of the EPC surface marker CD71 and the appearance of the erythroid markers CD235a and CD239, thereby inhibiting the differentiation of EPCs and accelerating the maturation of EPCs [139]. It is because HIF-1α promotes the expression of the erythroid surface markers CD71 and CD235a, so HIF-1α plays a vital role in fostering erythroid differentiation. Hypoxia increases the expression of GATA-1. Overexpression of GATA-1 increases HIF-1α in cord blood CD34^+^ and K562 cells. So GATA-1 is also required for normal erythropoiesis [140]. In addition, caspase-1 is necessary for HSPC bone marrow differentiation, and caspase-1 promotes erythroid differentiation by cleaving the major erythroid transcription factorGATA1 [141].

Supplementation with iron, folic acid, and vitamin C can promote erythroid differentiation [142,143]. The fetal hemoglobin inducer MS-275 to reactivate the fetal hemoglobin-producing γ-globin gene can encourage the production of hemoglobin and erythroid differentiation in K562 cells [144]. Splenectomy can also inhibit the expansion of EPCs, but its clinical efficacy is controversial [59]. The aryl hydrocarbon receptor (AHR) plays an essential role in mammalian embryonic development. Antagonism of AHR signaling enhances the production of human embryonic stem cell (hESC)-derived erythroid cells and promotes terminal erythroid differentiation [145].

**Figure 5:**
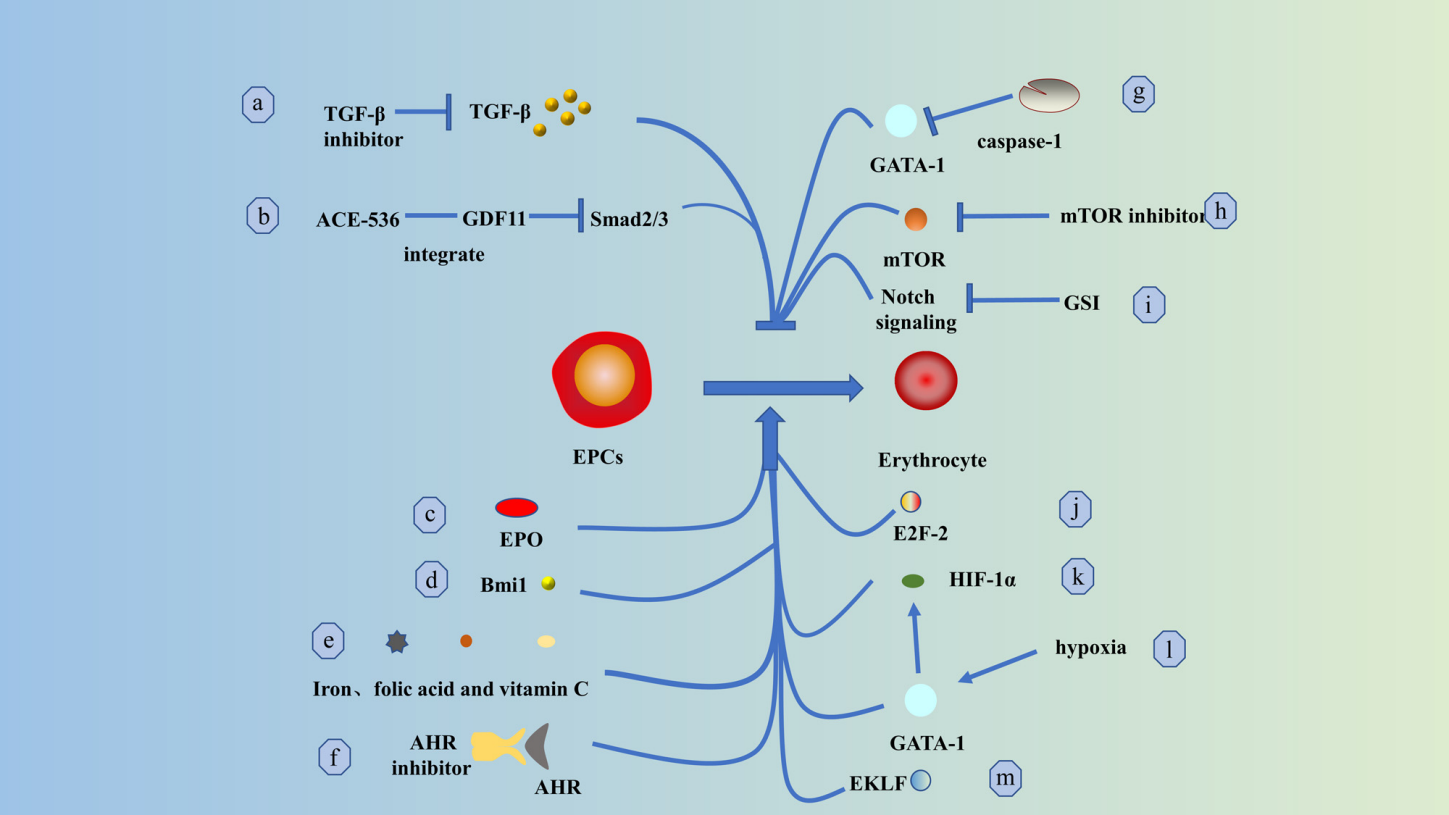
Critical modulations that promote differentiation of erythroid progenitors: (a) inhibition of TGF-β and (b) binding of GDF11 via ACE-536 effectively rescued differentiation arrest of EPCs; (c) EPO and (d) Bmi1 enhanced differentiation of EPCs; (e) iron, folic acid, and vitamin C also promoted differentiation of EPCs; (f) antagonism of AHR signaling improved hESC-derived erythrocyte production and enhanced terminal differentiation of EPCs; (g) caspase-1 promotes differentiation of EPCs by cleaving GATA1; (h) inhibition of mTOR signaling enhances maturation of EPCs; (i) inhibition of Notch signaling by GSI induces differentiation of EPCs and promotes hemoglobin production; (j) E2F-2 is expressed at high levels in (k). GATA-1 is essential for the differentiation and maturation of late EPCs; (l) hypoxia increases the expression of GATA-1 protein, and overexpression of GATA-1 increases the level of HIF-1α, which promotes the differentiation and maturation of EPCs; and (m) enhanced EKLF in late EKLF of EPCs may promote differentiation of terminal red lineage cells [161,162].

Adenovirus early region 2 binding factors (E2F-2), a transcription factor regulated by retinoblastoma, have a canonical function in promoting cell cycle progression [146,147]. In late erythroid maturation, high levels of E2F-2 expression promote nuclear cohesion and enucleation in terminal erythroid lineage cells [148]. Polycomb histone (PcG) is a crucial regulator of the terminal differentiation of HSCs [149–151]. The PcG gene BMI-1 regulates HSC self-renewal and promotes erythroid differentiation [152–154]. The mechanistic target of rapamycin (mTOR) is critical for cell growth (size) and proliferation [155]. Excessive activation of mTOR signaling interferes with cell cycle progression in Foxo3 mutant erythroid cells. Inhibiting mTOR signaling *in vivo* and *ex vivo* significantly enhanced Foxo3 mutant red lineage cells [156]. Because promotion and inhibition of erythroid cell proliferation have different effects on different diseases [47,157–160], further clinical studies are needed to investigate the appropriate therapeutic strategies (Figure 5 and Table 1).

**Table 1:** Regulatory pathways that promote the differentiation of EPCs

Mechanisms	Effects	Ref
TGF-β inhibitor	↑promote the differentiation of EPCs	[111–113]
ACE-536		[111]
EPO		[87,96,128]
PcG, Bmi1		[149–154]
Iron, folic acid, and vitamin C		[142,143]
AHR antagonist		[145]
Caspase-1		[141]
mTOR inhibitor		[155]
GSI		[137]
E2F-2		[146–148]
HIF-1α		[140]
Hypoxia		[139]
EKLF		[148]
GATA-1		[90,91,92,93]
MS-275		[144]
Resection of the spleen		[59]
EKLF/KLF1		[163]
Long non-coding RNAs		[109]
PPAR-α		[103]
RHEX		[104]

## 7 Conclusions

With the advancing research related to the role of EPCs in tumors, we are getting more explicit about the mechanisms by which EPCs affect tumors. EPCs are essential mechanisms of tumor growth, metastasis, and drug resistance. Further exploration of EPCs can help us gain insight into the immune microenvironment and thus identify potential targets for immunotherapy. Different subtypes of EPCs have different surface markers and expressions and play different roles in tumor immune evasion or progression. Moreover, high cell counts of EPCs always indicate poor prognosis and are also associated with tumor size and lymph node metastasis. Therefore, immunofluorescence screening of EPCs may be a novel clinical method to predict tumor recurrence. Clinical studies on EPCs are currently limited. Whether future therapeutic decisions can be made based on the expression of different phenotypes of EPCs still needs to be further explored. Previous studies have provided us with the thought that immunotherapy combined with radiotherapy and chemotherapy may be the most effective treatment modality. Besides, the treatment of cancer-related anemia is controversial. It will be a future research direction on how to treat anemia and improve the prognosis of oncology patients. As a result, addressing anemia and reducing EPCs’ pro-tumor function are crucial to improving the prognosis of cancer patients. Until now, that seems to be a promising immunotherapeutic strategy for patients with tumors combined with anemia. Finally, further information on artemisinin’s mechanism and signaling route is needed. The link between EPCs and malignancies and the TME are determined. This will open up new avenues for tumor diagnostics and therapy.
